# Supplementary material for: Chemical Analysis of Plasma-Activated Culture Media by Ion Chromatography
Source: Pharmaceuticals (Basel). 2025 Feb 1;18(2):199. doi: 10.3390/ph18020199 (PMC11858801; doi:10.3390/ph18020199)
Supplement: Supplementary file 1 [file pharmaceuticals-18-00199-s001.zip › pharmaceuticals-3368193-supplementary.pdf]

Table S1. PK parameters

Nitrite				
		T 1/2	*T max*	*C max*
**DMEM**	Vol 5 min 5	Missing	4320	13.51162665
	Vol 2 Min 5	1816,050	5	74.695
	Vol 5 Min 10	425,431	1440	37.861
	Vol 2 Min 10	1547.857	10	186.368
	Vol 5 Min 20	6523.652	20	128.487
	Vol 2 Min 20	Missing	2880	273.841
**McCOY**	Vol 5 min 5	1843.281	5	33.386
	Vol 2 Min 5	1405.518	5	83.925
	Vol 5 Min 10	Missing	10	0.217
	Vol 2 Min 10	Missing	2880	19.666
	Vol 5 Min 20	6470,349	20	140.242
	Vol 2	Missing	2880	202.441

Nitrate				
		T 1/2	*T max*	*C max*
**DMEM**	Vol 5 min 5	697.670	5	107.457
	Vol 2 Min 5	Missing	1440	10.834
	Vol 5 Min 10	Missing	0	0.245
	Vol 2 Min 10	Missing	2880	47.273
	Vol 5 Min 20	5110.991	20	82.478
	Vol 2 Min 20	1012.867	20	113.716
**McCOY**	Vol 5 min 5	975.842	5	34.402
	Vol 2 Min 5	1355.631	5	22.959
	Vol 5 Min 10	132840.137	10	1.115
	Vol 2 Min 10	Missing	4320	44.165
	Vol 5 Min 20	2569.699	20	99.226
	Vol 2 Min 20	Missing	2880	58.961

	Min 20			
**RPMI**	Vol 5 min 5	672.635	1440	0.239
	Vol 2 Min 5	Missing	1440	6.313
	Vol 5 Min 10	2976.537	1440	3.877
	Vol 2 Min 10	Missing	1440	6.104
	Vol 5 Min 20	Missing	20	0.032
	Vol 2 Min 20	876.807	1440	8.491

**RPMI**	Vol 5 min 5	Missing	1440	61.231
	Vol 2 Min 5	1103.839	5	64.033
	Vol 5 Min 10	718.449	0	59.953
	Vol 2 Min 10	Missing	4320	95.681
	Vol 5 Min 20	1945.511	0	59.953
	Vol 2 Min 20	missing	4320	95.666

In figure S1 is reported two chromatograms obtained from DMEM analysis at 24 and 72 hours. It’s evident that areas and concentration have decreased during time.

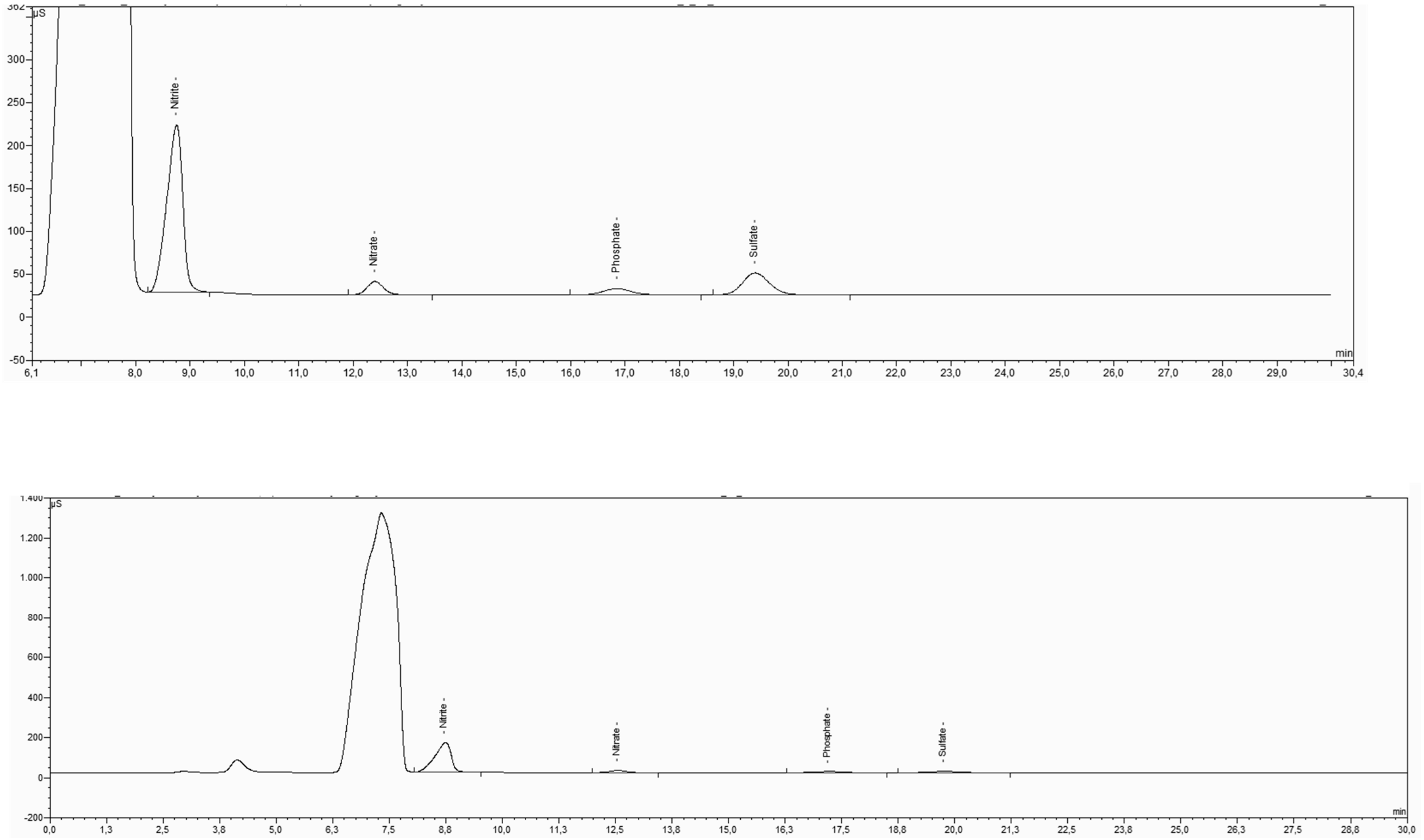

Figure S1: Chromatograms of DMEM at 24 and 72 hours.
